# Publisher’s Note: Cellular & Molecular Biology Letters, Volume 20

**DOI:** 10.1186/s11658-016-0999-4

**Published:** 2016-11-21

**Authors:** Cellular & Molecular Biology Letters

**Affiliations:** BioMed Central, Floor 6, 236 Gray’s Inn Road, London, WC1X 8HB UK

*Cellular & Molecular Biology Letters* launched with BioMed Central in January, 2016. Previously, its publishers were Walter De Gruyter in 2015, and Springer from 2006–2014. Content from 2015 (Volume 20) is available in the archive.

